# A Transcriptomic Analysis of Gonads from the Low-Temperature-Induced Masculinization of *Takifugu rubripes*

**DOI:** 10.3390/ani11123419

**Published:** 2021-11-30

**Authors:** He Zhou, Yuqing Sun, Xin Li, Ziyu Zhou, Kexin Ma, Wenxuan Guo, Yuting Liang, Xingyi Xie, Jingxian Zhang, Qian Wang, Yang Liu

**Affiliations:** 1 Key Laboratory of Mariculture, Agriculture Ministry, PRC, Dalian Ocean University, Dalian 116023, China; zhouhe@dlou.edu.cn (H.Z.); 17866543360@163.com (Y.S.); lixin949375269@163.com (X.L.); zzy980327@163.com (Z.Z.); makexin20211029@163.com (K.M.); gwxuan1999@163.com (W.G.); lyt18040108532@163.com (Y.L.); xiexingyi9909@163.com (X.X.); niuniudechuchu@163.com (J.Z.); 2 Key Laboratory of Marine Bio-Resources Sustainable Utilization in Liaoning Province’s University, Dalian Ocean University, Dalian 116023, China; 3 Key Lab of Sustainable Development of Marine Fisheries, Ministry of Agriculture, Yellow Sea Fisheries Research Institute, Chinese Academy of Fishery Sciences, Qingdao 266071, China; 4 Laboratory for Marine Fisheries Science and Food Production Processes, Pilot National Laboratory for Marine Science and Technology (Qingdao), Qingdao 266071, China

**Keywords:** transcriptomic, low-temperature, pseudo-male, qPCR, *Takifugu rubripes*

## Abstract

**Simple Summary:**

Our study analyzed the differentiation of transcriptomes in normal and sex-reverse *Takifugu rubripes*, and screened out 13 differentially expressed genes related to sex differentiation. This is the first report on the gonadal transcriptome of pseudo-males in *Takifugu rubripes*. Our results provide an important contribution to the molecular mechanism of masculinization in a cultured fish subject to low-temperature treatment.

**Abstract:**

The phenotypic sex of fish is usually plastic. Low-temperature treatment induces the masculinization of *Takifugu rubripes*, resulting in pseudo-males (PM) with the physiological sex of a male (M) and genetic sex of a female (F). For a comparison of gonadal transcriptomes, we collected gonads from three groups of *T. rubripes* (F, M, and PM) for high-throughput transcriptome sequencing. The results provided 467,640,218 raw reads (70.15 Gb) and a total of 436,151,088 clean reads (65.43 Gb), with an average length of 150 bp. Only 79 differentially expressed genes (DEGs) were identified between F and PM, whereas 12,041 and 11,528 DEGs were identified between F and M, and PM and M, respectively. According to the functional annotation of DEGs, 13 DEGs related to gonadal development were screened (*LOC101066759*, *dgat1*, *limk1*, *fbxl3*, *col6a3*, *fgfr3*, *dusp22b*, *svil*, *abhd17b*, *srgap3*, *tmem88b*, *bud4*, and *mustn10*) which might participate in formating PM. A quantitative PCR of the DEGs confirmed the reliability of the RNA-seq. Our results provide an important contribution to the genome sequence resources for *T. rubripes* and insight into the molecular mechanism of masculinization in a cultured fish subject to low-temperature treatment.

## 1. Introduction

Many economically important cultured fishes differ by sex in terms of their production performance, body size, growth rate, or age at sexual maturity [1]. The artificial control of fish sex, especially to select for best growth performance, is widely applied in aquaculture. Currently, at least 50 species of fish have been successfully induced to artificially reverse sex, including *Pelteobagrus fulvidraco* [2], *Dicentrarchus labrax* [3], *Scophthalmus maximus* [4], *Cyprinus* *carpio* [5], *Chlamys nobilis* [6], *Oreochromis niloticus* [7], and *Cynoglossus semilaevis* [8]. *Takifugu rubripes* is among the most commonly cultured fishes in China, Korea, and Japan; the annual production of *Takifugu rubripes* in 2019 was 9911 tons. Accordingly, it is a preferred aquaculture product and an important product for export in northern China. Although the growth rate of males and females of *T. rubripes* is similar, the meat quality of mature males is superior and the testis is considered a delicacy; therefore, the price of mature males is generally twice that of females [9]. The induction of sex reversal in fish by exposure to a low water temperature is safe and efficient; this method avoids the harm sometimes caused by traditional methods, including ultraviolet radiation and sex hormone supplementation [10]. The efficient production of greater proportions of male *T. rubripes* amounts to more profitable aquaculture.

A few studies have reported on *T. rubripes* masculinization. Hattori et al. [11] showed that the proportion of male *T*. *rubripes* juveniles could be significantly increased by low-temperature cultivation for 2–3 weeks post-hatching. Liu et al. [12] showed that low-temperature treatment effectively increased the proportion of males in *T. rubripes* family lines, with 15 °C being the optimum treatment temperature. Our team successfully produced an average proportion of 75% males using low-temperature treatment [13]. Zhou et al. [14] also performed comprehensive whole-genome methylation sequencing; analyses of the gonads of male, female, and pseudo-male *T*. *rubripes* revealed the highest methylated CpG content on chromosome 8 and the lowest on chromosome 5. However, to date, no reports of the transcriptome of pseudo-male *T. rubripes* have been published. Herein, gonads from low-temperature treated pseudo-males, normal females, and males were used for RNA sequencing (RNA-seq). The differentially expressed genes (DEGs) of each comparison were screened to explore the formation mechanism of pseudo-males. Our results lay a foundation for exploring the molecular mechanism of masculinization in *T. rubripes*.

## 2. Materials and Methods

### 2.1. Ethics Approval and Consent to Participate

The collection and handling of fish and experimental procedures were performed in accordance with the Guidelines for Experimental Animals of the Ministry of Science and Technology (Beijing, China), and approved by the Institutional Animal Care and Use Committee, IACUC of Yellow Sea Fisheries Research Institute, CAFS (Qingdao, China) (No. YSFRI-2021020).

### 2.2. Fish Sample Collection

The male parent is 51.3 cm and 3.42 kg, and the female parent is 42.4 cm and 1.98 kg. The sexual maturity season of *Takifugu rubripes* is around March. The fish samples used in this study were produced using the low-temperature treatment-induced masculinization of *T. rubripes* described in Zhou et al. [15]. At 50 days post-hatch (dph), we incubated them at 13 °C for 45 days; then we raised them at 21 ± 1 °C. Meanwhile, the control group was cultured at 21 ± 1 °C. At 230 days post-hatch, the caudal fins and gonads were dissected from the control groups of females (F) and males (M) and the treatment group of pseudo-males (PM). Part of the gonads was fixed in Bouin’s solution for 24 h and thereafter stored in 70% alcohol solution for physiological sex identification. The other part of the gonads was fixed in liquid nitrogen and stored at −80 °C for RNA-seq. The caudal fin was fixed with 95% alcohol for genetic sex identification.

### 2.3. Screening of Pseudo-Males

Screening for PM followed the method of Zhou et al. [15]. The phenotypic sex was identified by a histological analysis, and the genotypic sex was identified by an SNP analysis of the *amhr2* gene using the PCR method [16]. The results of the phenotypic sex identification and genetic sex identification were combined to screen for PM.

### 2.4. RNA Collection and Sequencing

The experiments were conducted with three biological replicates for each sex (i.e., samples from three individuals were mixed as one biological sample). The samples were sent to Sangon Biotech Co., Ltd. (Shanghai, China) for RNA-seq and data analysis. After quality control, the eukaryotic mRNA of the nine samples was enriched by magnetic beads affixed to oligo (dT), followed by paired-end sequencing, and the cDNA library was constructed according to the rule that the average insertion size of the cDNA library was 2 × 150 bp (150 paired-end runs).

### 2.5. Processing of Raw Reads

The raw reads obtained by sequencing from the Illumina HiSeq™ 2500 sequencing platform included some sequences with joints or of lower quality. To ensure the accuracy of the information in the analysis, filtering the original data and obtaining clean reads is necessary. FastQC 0.11.2 software (http://www.bioinformatics.babraham.ac.uk/projects/fastqc (accessed on 20 August 2019)) was employed to assess the quality of the raw reads. After quality evaluation, the data were trimmed using the Trimmomatic 0.36 tool [17] to improve accuracy and effectiveness. Data processing using the Trimmomatic included the following: obtaining clean reads from raw reads after the process of joint removal and low-quality data filtering. The high-quality reads were mapped to the reference genome of *T. rubripes* by HISAT2 2.1.0 [18].

### 2.6. Screening and Analysis of Differentially Expressed Genes (DEGs)

The open-source software StringTie 1.3.3b [19] and known gene models were used to evaluate gene expression. Read counts were positively correlated with gene length and sequencing depth and with the true expression level of the gene. The transcripts per million (TPM) normalization method was used to measure the proportion of a transcript in the RNA pool to make the estimated expression level of the gene comparable between different genes and experiments. After the gene expression data of each sample are obtained, they can be used to analyze the DEGs between different samples. The data were divided into three groups for comparison: F vs. M, F vs. PM, and M vs. PM. The difference in gene expression was analyzed by DESeq2 with a threshold of log_2_|fold-change| > 2, and a q-value < 0.05. Heatmaps were drawn using MeV. The DEGs were compared with the public databases Gene Ontogeny (GO) and the Kyoto Encyclopedia of Genes and Genomes (KEGG); the threshold was e ≤ le − 10. The functional enrichment was analyzed for sequence similarity alignment using the Basic Local Alignment Search Tool (BLASTX).

### 2.7. Quantitative PCR Verification

The reliability of the RNA-seq results was validated by qPCR; three DEGs were selected from the transcriptome sequencing results for qPCR verification (*mustn1*, *sox3*, and *zglp1*). Total RNA was reverse transcribed to first-strand cDNA using RevertAid™ Premium Reverse Transcriptase kits (#EP0733; Thermo Scientific, Waltham, MA, USA). The primers are listed in Table 1. *Β-actin* was used as a reference gene. The reaction system included 10 μL of SybrGreen qPCR Master Mix, 0.4 μL of primer R (10 μM), 0.4 μL of primer F (10 μM), 2 μL of template cDNA, and 7.2 μL of ddH2O. The thermal-cycling parameters were initiated at 95 °C for 3 min followed by 45 cycles of 95 °C for 7 s for denaturation, 57 °C for 10 s for annealing, and 72 °C for 15 s for an extension. The experiments were conducted with three biological replicates for each sample (i.e., samples from three individuals were mixed as one biological sample). The 2^−ΔΔCT^ method was used to analyze the expression levels of the target genes. All data were expressed as mean ± standard error (SE) and then tested for statistical significance using one-way ANOVA in IBM SPSS software version 25.0 (SPSS Inc., Chicago, IL, USA). *p* < 0.05 was considered statistically significant, and *p* < 0.01 was considered highly significant.

## 3. Results

### 3.1. Screening of Pseudo-Males

**Figure 1:**
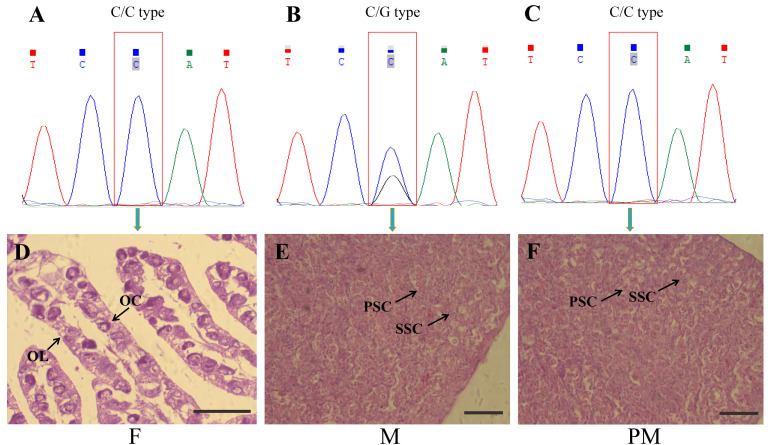
Genetic and phenotypic sex identification in *Takifugu rubripes*. Gene types: (**A**) female, (**B**) male, and (**C**) pseudo-male; cross-sections of (**D**) female ovary, (**E**) male testis, and (**F**) pseudo-male testis. OC, oocytes; OL, ovarian lamella; PSC, primary spermatocytes; SSC, secondary spermatocytes. Scale bars = 100 μm.

Combining the results of the genetic and physiological sex identification of the *T. rubripes*, samples with the physiological sex male and the genetic sex female were identified as pseudo-males. Figure 1A,D show the results of genetic and physiological sex identification for group F; Figure 1B,E show the results for group M; and Figure 1C,F show the results for group PM. The single nucleotide polymorphism at the *amhr2* locus was C/C in F and PM (Figure 1A,C), and in M it was C/G (Figure 1B). Histological sections of the gonads revealed the testis structure in groups M and PM (Figure 1E,F), and the ovary structure in group F (Figure 1D). Oocytes and ovarian lamella were observed in the female gonad (Figure 1D). Primary and secondary spermatocytes were observed in the male gonad (Figure 1E). The PM gonad showed characteristics of a male gonad, with primary and secondary spermatocytes observed (Figure 1F).

### 3.2. Data Statistics

A total of 467,640,218 raw reads (70.15 Gb) were obtained from *T. rubripes* M, F, and PM gonads by RNA sequencing. Subsequently, 436,151,088 clean reads (65.43 Gb) were obtained after filtering; the average yield was 93.28%, the total number of GC bases was 439,531,234, and the average GC content was 50.42%. Among the clean reads obtained, each sample had high percentages of Q20 and Q30 (Table 2). The uniquely mapped reads ranged from 91.19% to 93.35% (Table 3).

### 3.3. Sequencing Results and Interpretation of DEGs

**Figure 2:**
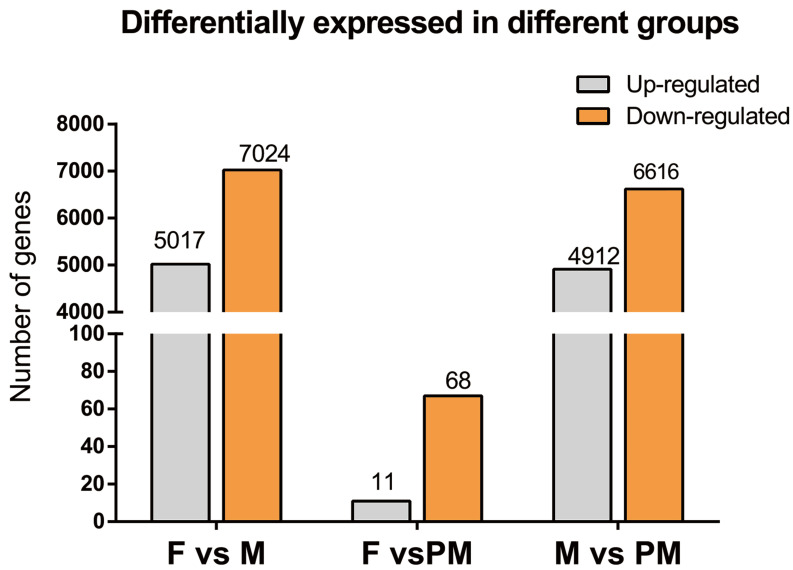
Histogram of differentially expressed genes in three groups of *Takifugu rubripes.* F, females; M, males; PM, pseudo-males. Gray indicates upregulated, and orange indicates downregulated.

The transcripts per million (TPM) normalization method was used to measure the proportion of a transcript in the RNA pool to make the estimated gene expression levels comparable between different genes and different experiments. After the gene expression data of each sample are obtained, they can be used to analyze the differential gene expression between two samples. We divided into three groups for comparison: F vs. M, F vs. PM, and M vs. PM. The difference in gene expression was analyzed by DESeq2. The default threshold for selecting genes with significant differences was |log_2_ fold-change| ≥ 1, *p* < 0.05. A total of 22,704 genes were expressed by TPM. There were 16,742; 20,652; and 16,600 genes in the female, male, and pseudo-male gonads, respectively. The number in F was close to that in PM, and the gene number in group M was much higher than in the other two groups. The number of DEGs between the F vs. M, F vs. PM, and M vs. PM comparisons were 12,041; 79; and 11,528, respectively (Figure 2). The number of DEGs was lowest between F and PM (11 F upregulated, 67 F downregulated), while the numbers were higher between F and M (5017 F upregulated, 7024 F downregulated) and between M and PM (4912 F upregulated, 6616 F downregulated). This may be because the PM group is genetically female, thereby resembling the F group in gene expression. Further studies on DEGs between F and PM groups are more likely to explain the occurrence of pseudo-males.

### 3.4. Gene Enrichment Analysis in Gene Ontology (GO)

**Figure 3:**
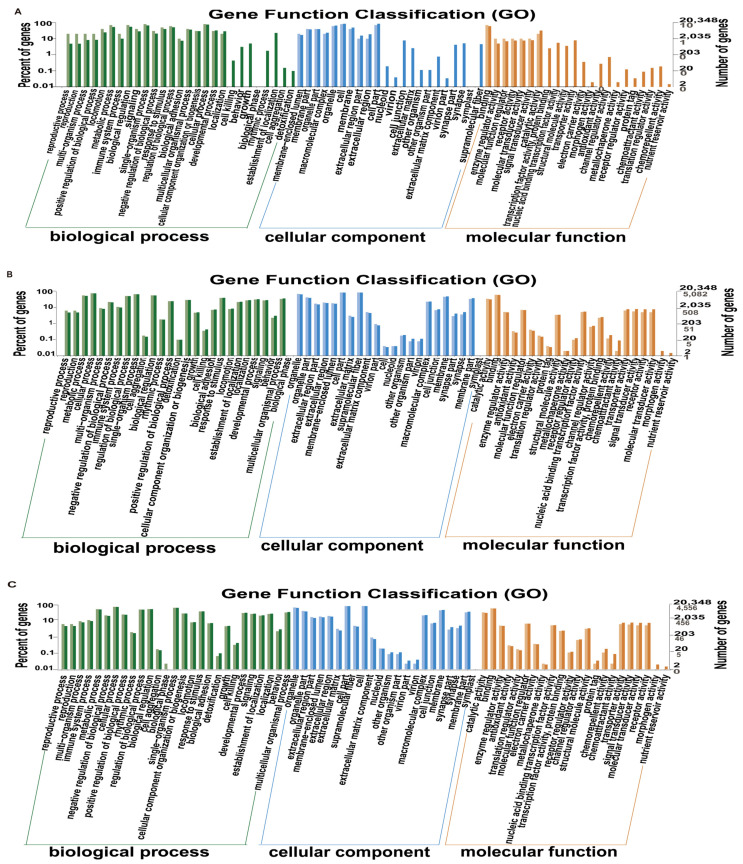
Classification histogram of Gene Ontology (GO) annotations of different genes in *Takifugu rubripes*. (**A**) Differential analysis of GO enriched terms of F vs. PM; (**B**) differential analysis of GO enriched terms of F vs. M; (**C**) differential analysis of GO enriched terms of M vs. PM. The horizontal axis is a functional classification, and the vertical axis is the number of genes in the classification (**right**) and their percentage in the total number of genes annotated (**left**). Pale colors represent differentially expressed genes, and dark colors represent all genes.

The number of genes annotated to the GO database by the F vs. PM, F vs. M, and M vs. PM groups was 20,348, including 16,260 molecular functions, 19,415 cellular components, and 17,382 biological processes (Figure 3). The number of DEGs from the F vs. PM, F vs. M, and M vs. PM groups to GO was 66; 10,861; and 10,416, respectively. The number of annotated genes to molecular function was 52, 8084, and 8297, accounting for 78.8%, 74.4%, and 79.7%, respectively, and included terms such as molecular function regulator, enzyme regulator activity, catalytic activity, chemoattractant activity, and chemorepellent activity. The number of annotated genes to the cellular component was 61, 10,372, and 9933, accounting for 92.4%, 95.5%, and 95.4%, respectively, and included terms such as extracellular region, virion part, virion, and other organisms. The number annotated to biological processes was 51, 9272, and 8899, accounting for 77.3%, 85.4%, and 85.4%, respectively, and included terms such as reproductive process, reproduction, detoxification, and cell killing. Notably, DEGs between F and PM were significantly enriched in reproduction-related pathways. We speculated that low-temperature treatment changed the expression level of genes related to gonad development in females, which promoted pseudo-male formation.

### 3.5. Gene Enrichment Analysis in the Kyoto Encyclopedia of Genes and Genomes (KEGG)

**Figure 4:**
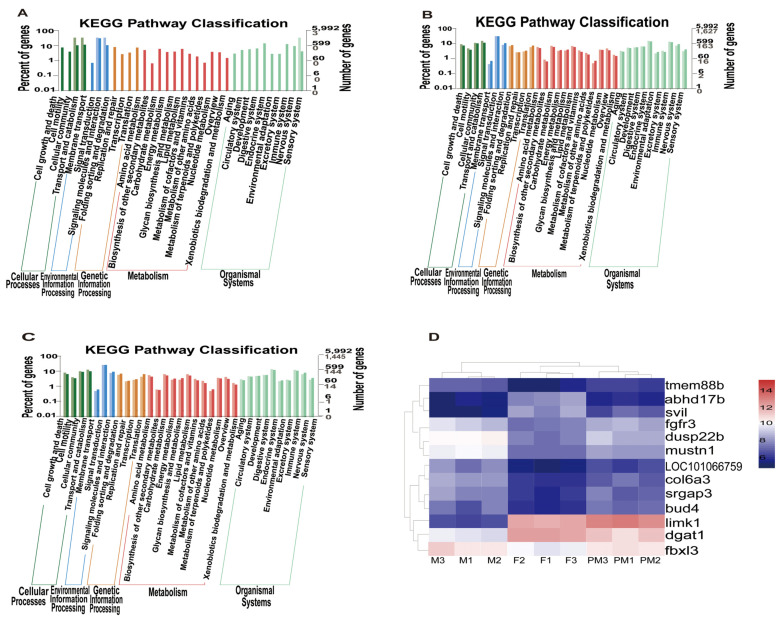
Classification histogram of KEGG annotations of different genes. (**A**) Differential analysis of KEGG enriched terms of F vs. PM; (**B**) differential analysis of KEGG enriched terms of F vs. M; (**C**) differential analysis of KEGG enriched terms of M vs. PM. The horizontal axis is a functional classification, and the vertical axis is the number of genes in the classification (**right**) and their percentage in the total number of genes annotated (**left**). Pale colors represent differentially expressed genes, and dark colors represent all genes. (**D**) Clustering thermogram of differentially expressed genes in three groups of *Takifugu rubripes*. F, females; M, males; PM, pseudo-males. Each row in the graph represents a gene, and the color represents the amount of gene expression in the sample. Red represents higher expression of the gene in the sample, and blue represents lower expression.

The number of DEGs in KEGG annotated by F vs. PM, F vs. M, and M vs. PM were 22, 3355, and 3219, respectively (Figure 4A–C). The results of the F vs. PM comparison were mainly related to the sensory system, signaling molecules and interaction, transport and catabolism, cellular community, and signal transduction. The results of the F vs. M comparison were mainly related to membrane transport, the metabolism of terpenoids and polyketides, and the biosynthesis of other secondary metabolites. The results of the M vs. PM comparison were mainly related to lipid metabolism, the metabolism of terpenoids and polyketides, and the sensory system. Therefore, we speculated that low-temperature treatment might affect the signaling molecules and interactions, and the transport and signal transduction pathways in females of *T. rubripes*, thus promoting sex reversal.

### 3.6. Clustering and Functional Analysis of DEGs

The results of the cluster analysis of DEGs for nine gonad samples (from F vs. PM) are shown in Figure 4D. As depicted in the figure, the PM and F samples are a branch; in accordance with the branch of the sample, we found that the gene expression of PM was mostly close to that of F, but a small part was close to that of M. Exploring the DEGs of PM and F is helpful for clarifying the mechanism of PM. According to this result and the functional annotation of DEGs, 13 DEGs related to gonadal development were screened (*LOC101066759*, *dgat1*, *limk1*, *fbxl3*, *col6a3*, *fgfr3*, *dusp22b*, *svil*, *abhd17b*, *srgap3*, *tmem88b*, *bud4*, and *mustn1*), and combined with the above results, we speculated that these genes might be related to PM occurrence.

### 3.7. Analysis of Quantitative PCR Results

**Figure 5:**
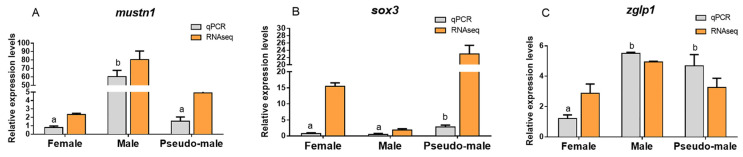
Comparison of gene expression analysis using qPCR and RNA-seq. (**A**) *mustn1*, (**B**) *sox3*, and (**C**) *zglp1* gene expressions in three groups of *Takifugu rubripes*. Gray indicates qPCR, and orange indicates RNA-seq.

The accuracy of RNA-seq was verified by comparing the expression of genes obtained by qPCR with that of RNA-seq. We set the female as calibration organization. Figure 5A–C shows histograms of qPCR and RNA-seq expressions of the *mustn1*, *sox3*, and *zglp1* genes. The expression trends of qPCR and RNA-seq were similar, which indicated that the results of RNA-seq were accurate.

## 4. Discussion

Sex is a product of evolution and the driving force for the continuous development of biological evolution. Therefore, exploring sex-determination mechanisms has always been among the most attractive and popular fields in life-science research [20]. In addition to genetic factors, a large amount of available data suggests that external environmental factors, and unknown factors, influence the sex of fish offspring, especially temperature [21]. Temperature-induced sex reversal has been successful for nearly 20 species of fish, including *Ictalurus punctatus*, *Paralichthys olivaceus*, *Cynoglossus semilaevis*, and *Atherina bleekeri* [22,23]. In recent years, our team obtained a 75% portion of male *T. rubripes* using low-temperature treatment [13,15]. This simple technology results in economic benefits for aquaculture and provides valuable information for exploring the molecular mechanism of sex differentiation and determination.

Studies on sex differentiation and determination in fish have mainly focused on single-gene function. Genes related to sex determination (e.g., *cyp19a1a* and *amh*) and epigenetic regulators appear to be downstream factors in the environmental sex reversal (ESR)-related pathways. Therefore, the identification of more upstream genes responding to the change in temperature is critical to further our understanding of ESR. The emergence of the transcriptome analyses can serve as a powerful tool to identify potential upstream regulators [24]. RNA-seq is widely used in many biological fields, including immunity, metabolism, toxicology, and the development and evolution of fish. Some studies have reported on sex determination and differentiation transcriptome analysis in fish, including the *Xiphophorus* species [25], *Oncorhynchus mykiss* [26], and *Misgurnus anguillicaudatus* [27,28]. Díaz et al. [3] found that results with *Dicentrarchus labrax* were useful for comparing the effects of heat on the behavior of cognate genes related to sex differentiation. Robledo et al. [4] profiled several genes (*cyp19a1a*, *vasa*, and *amh*) involved in sex differentiation and found specific temperature effects on gene expression in *Scophthalmus maximus*. Jia et al. [5] found a higher number of DEGs from an undifferentiated juvenile ovary (vs. comparisons with the transcriptome of an adult ovary), which were assigned to reproduction terms, in *Cyprinus carpio*. Furthermore, their investigation revealed that DEGs identified from an undifferentiated juvenile-ovary analysis were enriched in several important functional pathways, including Fanconi anemia and the Notch signaling pathway. Shi et al. [6] suggested highly conserved sex reversal/differentiation with diverged regulatory pathways during *Chlamys nobilis* evolution. Sun et al. [7] demonstrated that high-temperature treatment caused a delay in spermatogenesis in heat-induced neo-males of Nile tilapia. The results of Liu et al. [8] indicated that DNA methylation, which regulates the expression of *cyp19a1a*, is the mechanism for temperature-induced masculinization in *Cynoglossus semilaevis*.

The present study used RNA-seq to analyze three groups (F, M, and PM) of *T. rubripes* gonadal transcriptome, wherein only the PM group had been treated with low-temperature incubation early in the experiment. Notably, GO analysis revealed a significant influence on the reproductive process and reproduction; therefore, we suspect that the presence of DEGs in F vs. PM influenced the gonad development of F, leading to a transformation into PM. Combined with the results of KEGG, we hypothesized that the early exposure of *T. rubripes* to a low temperature leads to the differential expression of signaling molecules and their interactions, transport and catabolism, cell communities, and genes related to signal transduction; the related function would then become abnormal, resulting in pseudo-males. According to the overall results of GO and KEGG analysis, the expressed genes between groups F and PM were similar in number and type. This may be because the genetic sex of PM is female and the physiological sex is male, which is close to F in genetic distance. Therefore, an in-depth study of the DEGs between F and PM could likely explain the mechanism resulting in the occurrence of pseudo-males.

To explore the difference in gonadal development between F and PM, the functional annotation GO, KEGG analyses, and chromosome location of DEGs were combined for analysis (Table S1), and 13 DEGs were screened out (Table 4). The GO and KEGG analysis indicated that *LOC101066759* and *dgat1* were mapped to a biological regulation pathway; *dgat1* is widely present in body tissues but is mainly expressed in testis, fat, and the mammary gland. As a key enzyme that catalyzes the synthesis of triglycerides, it has recently been implicated in causing a rare nutritional and digestive disease [29]. Xia et al. [30] found that *dgat1* expression is associated with the clinical phenotype of ovarian cancer. We hypothesized that the differential expression of *dgat1* between F and PM might affect the synthesis of triglyceride, leading to abnormal energy sources during gonad development and then to PM generation. Sakae et al. found that inhibiting fatty acid synthesis also caused sex reversal in Olyzias latipes [31]. In our study, we found that the *dgat1* gene is a differential gene between F and PM, and *dgat1* is related to fatty acid synthesis. Therefore, we speculated that the *dgat1* gene affects the gonadal development of PM by affecting fatty acid synthesis. The GO and KEGG analysis indicated that the *limk1* gene was mapped to a biological process regulation, development process, and growth pathway. The LIM kinase family, a serine/threonine protein kinase present in eukaryotes, mainly includes *limk1* and *limk2*. As cofilin phosphatases, LIM kinases can regulate the dynamics of actin and microtubule by phosphorylating ser3 of cofilin, in anticipation of the proliferation and motion of cells [32]. The differential expression of the *limk1* gene in F and PM might lead to abnormal cell proliferation and motility, and thereby to the transformation of F into PM.

Regarding the location of genes on chromosomes, we found that the genes *fbxl3* and *col6a3* were located on chromosome 1 and expressed in the same subgroup (Figure 3). Studies have shown that *fbxl22* is in the same gene family as *fbxl3*, and it is essential for the maintenance of normal contractile function in vivo [33,34]. Research has shown that *col6a3* is associated with connective tissue growth and remodeling [35]. The *fgfr3* gene is located on chromosome 2, and *dusp22b* is located on chromosome 22; Figure 3 shows that these two genes were likewise expressed in the same subgroup. The expression of *dusp22b* is related to the estrogen receptor [36]. In one study, the deletion of the *fgfr3* gene reversed the sex of male mice, and *fgfr3* was widely expressed during the embryonic testis development; these findings suggested that the gene is related to the formation and development of the testis [37]. We speculate that the low-temperature treatment resulted in the abnormal expression of this gene in F, which promoted the development of testis and thus the transformation of F into PM. The gene *svil* was located on chromosome 5, and the location of *abhd17b* was unknown; again, these two genes were expressed in the same subgroup (Figure 3).

**Table 4:** Functional annotation and chromosome localization of differentially expressed genes (DEGs) between female and pseudo-male *Takifugu rubripes*.

Gene ID	Annotation	Gene Name	Location in Chromosome
fbxl3	F-box and leucine rich repeat protein 3	*fbxl3*	Chr 1:11,900,578–11,913,090
LOC101075798	collagen alpha-3(VI) chain-like	*col6a3*	Chr 1:22,703,149–22,724,053
LOC101063860	fibroblast growth factor receptor 3-like	*fgfr3*	Chr 2:10,771,162–10,816,015
LOC105416485	supervillin-like	*svil*	Chr 5:10,546,661–10,552,527
LOC101061422	LIM domain kinase 1-like	*limk1*	Chr 11:3,287,016–3,309,899
dgat1	diacylglycerol O-acyltransferase 1	*dgat1*	Chr 12:9,699,595–9,709,017
srgap3	SLIT-ROBO Rho GTPase activating protein 3	*srgap3*	Chr 19:4,938,744–4,975,164
tmem88b	transmembrane protein 88B	*tmem88b*	Chr 19:5,227,381–5,231,818
LOC101077437	bud site selection protein BUD4-like	*bud4*	Chr 19:5,911,121–5,918,371
mustn1	musculoskeletal, embryonic nuclear protein 1	*mustn1*	Chr 19:4,520,735–4,521,811
LOC101072874	dual specificity protein phosphatase 22-B-like	*dusp22b*	Chr 22:145,042–148,673
LOC101078060	alpha/beta hydrolase domain-containing protein 17B	*abhd17b*	unknown
LOC101066759	immune-type receptor		unknown

According to gene annotation, the *svil* gene was related to supervillin, and the *abdh* gene family contained a wide range of substrate-specific enzymes, which are related to catalytic activity. The genes *srgap3*, *tmem88b*, *bud4*, and *mustn1* are located on chromosome 19, which is the sex chromosome of *T. rubripes*. The *srgap3* gene is an important modulator of actin cytoskeletal dynamics and has an important influence on a range of neurodevelopmental processes [38]. For instance, the *srgap3*-mediated reorganization of the actin cytoskeleton is crucial for the normal development of dendritic spines, and the loss of *srgap3* leads to abnormal synaptic activity in mice [39]. *mustn1* is a small nuclear protein involved in the development and regeneration of the musculoskeletal system. Three genes related to gonadal development or located on sex chromosome 19 were selected to assess the reliability of RNA-seq by qPCR. The gene *mustn1* is located on sex chromosome 19 and was upregulated in M but downregulated in F and PM; therefore, we speculate that *mustn1* is probably involved in testis development. In addition, Abdelmoneim et al. found that the *slc9a3* gene was male- or testicular-specific in Oryzias latipes [40]. In our study, we found that the *slc1a3* gene was significantly different between F and PM. In zebrafish, *cldn19* expression levels were highly affected by estrogen exposure, rising on average in primary follicles [41]. Molecular changes occurring during mammalian oocyte maturation are partly regulated by cytoplasmic polyadenylation (CP) and affect oocyte quality. Reyes et al. [42] found that the *cldn19* gene was upregulated which can influence female (or ovarian-specific) and steroid-sensitive expression by RNA sequencing. In our study, we found that the *cldn30c* gene was significantly different between F and PM. Pan et al. [43] found that the *nectin* gene is involved in spermatogenesis, and *nr0b1* belongs to the nuclear receptor (NR) superfamily. It plays critical roles in sex determination, sex differentiation, and gonadal development in mammals [44]; we also found that the *nectin* and *nr0b1* genes belong to the DEGs between F and PM. We speculate that the DEGs between F and PM affect phenotypic sex differentiation and the expression of gonadal development-related genes in various ways, thus affecting gonadal development and leading to the production of pseudo-males.

## 5. Conclusions

This is the first study about the transcriptome of pseudo-male *T. rubripes* using the RNA-seq method to analyze the gene expression of the transcriptome of PM gonads. Thirteen DEGs were screened (*LOC101066759*, *dgat1*, *limk1*, *fbxl3*, *col6a3*, *fgfr3*, *dusp22b*, *svil*, *abhd17b*, *srgap3*, *tmem88b*, *bud4*, and *mustn10*) and were related to the gonadal development of pseudo-males. These results provide rich genome sequence resources for future research. We suspect that changes in the expression levels of these genes, as caused by the low-temperature treatment of the *T. rubripes*, are responsible for the production of pseudo-males.

## Figures and Tables

**Table 1:** List of qPCR primer sequences.

Gene Name	Forward Primer (F)/Reverse Primer (R) (5′–3′)	
*mustn1*	F	CGCCCAGAGGTAAGGAAAGA
	R	TCTCTCGCATTCCTCCATTACT
*zglp1*	F	AGGAGTTCAGCAGAAACGGAG
	R	GCAACGACAGGTTCGCATT
*sox3*	F	AACAACAGCAGCAACGAGGAT
	R	CGTCGGTCAGAAGTTTCCAGT

**Table 2:** Sample sequencing output data-quality assessment form.

Sample	Raw Data	Q20 (%)	Q30 (%)	GC (%)	Clean Reads	Clean/Raw (%)
PM_1	49,139,712	97.10%	92.89%	51.59%	45,934,400	93.48%
PM_2	50,438,140	97.02%	92.71%	51.81%	47,102,818	93.39%
PM_3	50,378,422	97.36%	93.44%	50.58%	47,490,250	94.27%
F_1	54,330,568	97.06%	92.81%	52.03%	50,886,434	93.66%
F_2	53,706,244	97.05%	92.80%	52.53%	50,073,694	93.24%
F_3	53,883,284	97.00%	92.67%	52.07%	50,106,682	92.99%
M_1	59,716,736	97.01%	92.92%	48.48%	55,127,742	92.32%
M_2	50,834,854	97.37%	93.59%	47.57%	47,324,666	93.09%
M_3	45,212,258	97.25%	93.35%	47.08%	42,104,402	93.13%

**Table 3:** Results of comparison with the reference genome.

Sample	Total Reads	Total Mapped	Multiple Mapped	Uniquely Mapped
PM_1	45,934,400 (100.00%)	41,885,381 (91.19%)	3,352,916 (7.30%)	38,532,465 (83.89%)
PM_2	47,102,818 (100.00%)	43,676,065 (92.72%)	3,219,480 (6.84%)	40,456,585 (85.89%)
PM_3	47,490,250 (100.00%)	43,686,271 (91.99%)	3,200,997 (6.74%)	40,485,274 (85.25%)
F_1	50,886,434 (100.00%)	46,897,430 (92.16%)	3,875,948 (7.62%)	43,021,482 (84.54%)
F_2	50,073,694 (100.00%)	46,745,301 (93.35%)	3,348,249 (6.69%)	43,397,052 (86.67%)
F_3	50,106,682 (100.00%)	46,262,973 (92.33%)	3,223,857 (6.43%)	43,039,116 (85.89%)
M_1	55,127,742 (100.00%)	50,553,238 (91.70%)	2,220,041 (4.03%)	48,333,197 (87.67%)
M_2	47,324,666 (100.00%)	43,389,429 (91.68%)	2,050,256 (4.33%)	41,339,173 (87.35%)
M_3	42,104,402 (100.00%)	38,849,353 (92.27%)	1,651,395 (3.92%)	37,197,958 (88.35%)

## Data Availability

The transcriptome data used in this study have been uploaded to the NCBI Sequence Read Archive (SRA) with accession number PRJNA591733.

## Supplementary Materials

The following are available online at https://www.mdpi.com/article/10.3390/ani11123419/s1, Table S1: Functional annotation and chromosome localization of some differentially expressed genes (DEGS) in Takifugu rubripes between female and pseudo-males.
