# Supplementary material for: A Transcriptomic Analysis of Gonads from the Low-Temperature-Induced Masculinization of Takifugu rubripes
Source: Animals (Basel). 2021 Nov 30;11(12):3419. doi: 10.3390/ani11123419 (PMC8697924; doi:10.3390/ani11123419)
Supplement: Supplementary file 1 [file animals-11-03419-s001.zip › animals-1464654-supplementary.pdf]

Supplementary Table S1. Functional annotation and chromosome localization of some differentially expressed genes (DEGS) in *Takifugu rubripes* between female and pseudo-males.

Gene ID	Annotation	Gene name	Location in chromosome
fbxl3	F-box and leucine rich repeat protein 3	*FBXL3*	Chr 1:11,900,578–11,913,090
nr0b1	nuclear receptor subfamily 0 group B member 1	*NR0B1*	Chr 1:12,437,109–12,438,835
LOC105416978	WAP, Kazal, immunoglobulin, Kunitz and NTR domain-containing protein 2-like	*WFIKKN2*	Chr 1:13,682,155–13,685,647
ackr3	atypical chemokine receptor 3	*ACKR3*	Chr 1:13,864,865–13,870,879
col6a3	collagen type VI alpha 3 chain	*COL6A3*	Chr 1:22,669,260–22,695,944
LOC101075798	collagen alpha-3(VI) chain-like	*COL6A3*	Chr 1:22,703,149–22,724,053
LOC101063860	fibroblast growth factor receptor 3-like	*FGFR3*	Chr 2:10,771,162–10,816,015
LOC105418633	ncRNA		Chr 2:4,739,660–4,743,482
rsad2	radical S-adenosyl methionine domain containing 2	*RSAD2*	Chr 2:6,108,939–6,111,713
pld4	phospholipase D family member 4	*PLD4*	Chr 2:8,476,160–8,479,654
LOC101075439	SLIT-ROBO Rho GTPase-activating protein 3-like	*SRGAP3*	Chr 3:215,649–237,773
LOC105419325	ncRNA		Chr 3:464,597–466,688
LOC101079354	neural cell adhesion molecule L1-like protein	*CHL1*	Chr 3:5,143,368–5,174,410
LOC101077094	rho guanine nucleotide exchange factor 3-like	*ARHGEF3*	Chr 3:8,046,651–8,051,655
col21a1	collagen type XXI alpha 1 chain	*COL21A1*	Chr 4:11,688,556–11,706,544
mir202	ncRNA	*MIR202*	Chr 4:249,349–249,433
LOC105416485	supervillin-like	*SVIL*	Chr 5:10,546,661–10,552,527
LOC101067318	heat shock 70 kDa protein 1	*HSPA1*	Chr 5:10,973,872–10,977,016
LOC101064467	perforin-1-like	*PRF1*	Chr 5:11,206,761–11,210,230
il2rb	interleukin 2 receptor subunit beta	*IL2RB*	Chr 5:3,632,230–3,637,835
LOC101062830	sphingosine kinase 1-like	*SPHK1*	Chr 5:4,360,320–4,374,553
LOC101062169	unknown		Chr 5:5,733,708–5,736,498
LOC101065649	otopetrin-2-like	*OTOP2*	Chr 5:7,661,198–7,665,013
LOC101065744	histone-lysine N-methyltransferase Smyd1-like	*SMYD1*	Chr 6:866,552–871,235
LOC101064911	cornifelin homolog	*CNFN*	Chr 7:1,138,094–1,140,239
LOC101071122	tumor necrosis factor receptor superfamily member 5-like	*CD40*	Chr 7:13,468,248–13,475,303
gpbar1	G protein-coupled bile acid receptor 1	*GPBAR1*	Chr 8:5,790,014–5,792,536
LOC101061862	C-C motif chemokine 14-like	*CCL14*	Chr 8:8,718,825–8,719,852

serpine1	serpin family E member 1	*SERPINE1*	Chr 8:9,222,652–9,225,815
LOC105416957	non-muscle caldesmon-like	*CALD1*	Chr 9:11,397,666–11,402,034
met	MET proto-oncogene, receptor tyrosine kinase	*MET*	Chr 9:12,381,702–12,429,720
LOC101061422	LIM domain kinase 1-like	*LIMK1*	Chr 11:3,287,016–3,309,899
cd3g/d	CD3 gamma/delta	*CD3G*	Chr 11:413,845–415,448
caln1	calneuron 1	*CALN1*	Chr 11:9,347,047–9,375,748
dtnbp1	dystrobrevin binding protein 1	*DTNBP1*	Chr 12:1,779,609–1,820,107
dgat1	diacylglycerol O-acyltransferase 1	*DGAT1*	Chr 12:9,699,595–9,709,017
LOC101064923	haptoglobin-like	*HP*	Chr 13:13,695,708–13,718,695
map7d2	MAP7 domain containing 2	*MAP7D2*	Chr 15:10,301,207–10,308,869
LOC105417502	nectin-4-like	*NECTIN4*	Chr 15:11,459,538–11,464,121
cd248	CD248 molecule	*CD248*	Chr 15:2,161,247–2,165,880
LOC105417434	capZ-interacting protein-like	*RCSD1*	Chr 15:2,302,972–2,309,906
cldn30c	claudin-4	*CLDN30C*	Chr 15:6,647,585–6,648,912
LOC101061966	mitochondrial uncoupling protein 2-like	*UCP2*	Chr 15:8,994,315–8,998,438
LOC105417598	ncRNA	—	Chr 16:10,246,201–10,251,315
tmc8	transmembrane channel like 8	*TMC8*	Chr 17:2,007,268–2,012,523
chrm2	cholinergic receptor muscarinic 2	*CHRM2*	Chr 18:3,294,570–3,339,752
srgap3	SLIT-ROBO Rho GTPase activating protein 3	*SRGAP3*	Chr 19:4,938,744–4,975,164
tmem88b	transmembrane protein 88B	*TMEM88B*	Chr 19:5,227,381–5,231,818
LOC101077437	bud site selection protein BUD4-like	*BUD4*	Chr 19:5,911,121–5,918,371
mustn1	musculoskeletal, embryonic nuclear protein 1	*MUSTN1*	Chr 19: 4,520,735–4,521,811
LOC101077887	zinc finger protein Gfi-1-like	*GFI1*	Chr 20:1,311,283–1,319,881
LOC101070379	FYN-binding protein-like	*FYB1*	Chr 20:10,266,285–10,277,093
pip5k1c	phosphatidylinositol-4-phosphate 5-kinase type 1 gamma	*PIP5K1C*	Chr 20:3,987,156–4,013,334
rasgef1b	RasGEF domain family member 1B	*RASGEF1B*	Chr 21:3,141,576–3,158,209
LOC101066967	excitatory amino acid transporter 1-like	*SLC1A3*	Chr 21:8,284,967–8,296,477
LOC101072874	dual specificity protein phosphatase 22-B-like	*DUSP22B*	Chr 22:145,042–148,673
LOC105418089	ncRNA	—	Chr 22:2,120,904–2,122,675
aire	autoimmune regulator	*AIRE*	Chr 22:6,678,235–6,681,583
LOC101069795	GTPase IMAP family member 8	*GIMAP8*	unknown
LOC105419077	protein ALEX-like	*GNAS*	unknown
LOC101078060	alpha/beta hydrolase domain-containing protein 17B	*ABHD17B*	unknown
LOC105418189	abhydrolase domain-containing protein DDB_G0269086-like	*DDB_G0269086*	unknown
LOC101072085	alpha-2-macroglobulin-like	*A2M*	unknown
LOC101071768	ceramide kinase-like	*CERK*	unknown
LOC101063178	DENN domain-containing protein 1C-like	*DENND1C*	unknown

LOC101067517	abhydrolase domain-containing protein 8-like	*ABHD8*	unknown
dok6	docking protein 6	*DOK6*	unknown
LOC101068432	occludin-like	*OCLN*	unknown
LOC105419207	ncRNA	—	unknown
LOC105419008	ncRNA	—	unknown
LOC105419196	ncRNA	—	unknown
LOC105419570	ncRNA	—	unknown
LOC105419372	ncRNA	—	unknown
LOC105418439	ncRNA	—	unknown
LOC101063704	immune-type receptor	—	unknown
LOC101063320	immune-type receptor	—	unknown
LOC101063762	immune-type receptor	—	unknown
LOC101066759	immune-type receptor	—	unknown
LOC105418188	uncharacterized	—	unknown
